# Adenosine diphosphate stimulates VEGF-independent choroidal endothelial cell proliferation: A potential escape from anti-VEGF therapy

**DOI:** 10.1073/pnas.2418752122

**Published:** 2025-01-21

**Authors:** Nilima Biswas, Tommaso Mori, Naresh Kumar Ragava Chetty Nagaraj, Hong Xin, Tanja Diemer, Pin Li, Yongxuan Su, Carlo Piermarocchi, Napoleone Ferrara

**Affiliations:** ^a^Department of Pathology, University of California San Diego, La Jolla, CA 92093; ^b^Department of Chemistry and Biochemistry, University of California San Diego, La Jolla, CA 92093; ^c^Department of Physics and Astronomy, Michigan State University, East Lansing, MI 48824; ^d^Department of Ophthalmology, University of California San Diego, La Jolla, CA 92093

**Keywords:** endothelial cells, age-related macular degeneration, angiogenesis, metabolism, platelets

## Abstract

We hypothesized that a strategy employing tissue-specific endothelial cells (EC) might facilitate the identification of tissue- or organ-specific vascular functions of ubiquitous metabolites. An unbiased approach was employed to identify water-soluble small molecules with mitogenic activity on choroidal EC. We identified adenosine diphosphate (ADP) as a candidate, following biochemical purification from mouse EL4 lymphoma extracts. ADP stimulated the growth of bovine choroidal EC (BCEC) and other bovine or human eye-derived EC. ADP induced rapid phosphorylation of extracellular signal-regulated kinase in a dose- and time-dependent manner. ADP-induced BCEC proliferation could be blocked by pretreatment with specific antagonists of the purinergic receptor P2Y1 but not with a vascular endothelial growth factor (VEGF) inhibitor, indicating that the EC mitogenic effects of ADP are not mediated by stimulation of the VEGF pathway. Intravitreal administration of ADP expanded the neovascular area in a mouse model of choroidal neovascularization. Single-cell transcriptomics from human choroidal datasets show the expression of P2RY1, but not other ADP receptors, in EC with a pattern similar to VEGFR2. Although ADP has been reported to be a growth inhibitor for vascular EC, here we describe its growth-stimulating effects for BCEC and other eye-derived EC.

## Significance

Using a direct biochemical approach, we identified adenosine diphosphate (ADP) as an endothelial cell (EC) mitogen. ADP is a ubiquitous metabolite, mostly produced by platelets; its effects on EC remain unclear. We characterized ADP's bioactivity on eye-derived EC. The effects were mediated by one of its purinergic receptors, P2Y1. Single-cell transcriptomics from human choroidal datasets show a strong expression of P2RY1 in EC. ADP administration stimulated angiogenesis in a mouse model of choroidal neovascularization. We hypothesize that ADP, when released by platelets at sites of endothelial damage, may stimulate angiogenesis that is not blocked by anti-vascular endothelial growth factor (VEGF) agents; thus, it may mediate escape from anti-VEGF therapy.

Angiogenesis plays an important role in many physiological and pathological conditions, and pro- and antiangiogenic therapies have been developed to combat different disease processes (1, 2). The key role of the vascular endothelial growth factor (VEGF) pathway in normal and pathological angiogenesis has been established by extensive preclinical and clinical studies (3, 4). Anti-VEGF agents have been FDA-approved for the treatment of several malignancies and for ocular neovascular disorders including age-related macular degeneration (AMD) (5). AMD is the leading cause of vision loss in the elderly, affecting many people worldwide (6). Early-stage AMD is characterized by “drusen” of various sizes and numbers and by abnormalities of the retinal pigment epithelium (RPE). Advanced AMD can be “neovascular” or “atrophic” (geographic atrophy) (6). Although the introduction of VEGF inhibitors has enabled major advances in the treatment of neovascular AMD, not all patients respond to anti-VEGF agents and thus there is still a need to discover additional therapeutic targets (5).

Stimulating angiogenesis in principle could be beneficial in ischemic disorders such as coronary or limb ischemia (7). However, in spite of promising results in animal models, human trials testing several angiogenic factors so far have been largely unsuccessful (4, 8). Thus, there is still a need to discover additional proangiogenic factors or pathways to treat diseases where stimulating angiogenesis could be beneficial.

In tumors, angiogenesis facilitates rapid tumor growth and metastasis (9). Tumor angiogenesis is determined by the coordinated activation of numerous signaling pathways, cross talk between EC, and other cell types in the tumor microenvironment (10). Recently, EC metabolism has been explored as a possible target for inhibiting angiogenesis (11). Indeed, cancer cells exhibit a distinct metabolic phenotype: promotion of glycolytic activity plus down-regulation of the TCA cycle and oxidative phosphorylation, called the Warburg effect (12). Thus, metabolism has been a focus of research aiming to elucidate key mechanisms underlying cancer growth; several endogenous metabolites (oncometabolites) that initiate or sustain tumor growth and metastasis have been identified (13, 14). Among metabolites, purines and pyrimidines have received a great deal of attention (15, 16). Indeed, purinergic signaling is involved in a variety of important pathophysiological processes such as cell survival, proliferation, differentiation, and motility (17). In the subretinal space, the purinergic pathways mediate the communication between the retina and RPE, influence the degeneration of the injured and diseased retina, and also protect the retinal tissue from degeneration (18, 19).

Since metabolism in pathological conditions may have some unique features, this study aimed to reveal metabolites as possible therapeutic targets. In search of a novel small-molecule regulator of EC growth, we investigated different tumor cell lines, leading to the identification of adenosine 5’-diphosphate (ADP) as a mitogen for bovine choroidal endothelial cells (BCEC). Although ADP has been characterized as an EC growth inhibitor for macrovascular EC such as bovine aortic EC (BAEC), human aortic EC (HAEC), and human umbilical vein EC (HUVEC) (20), here we report that ADP stimulates proliferation of a microvascular EC type in vitro through activation of P2Y1. A similar discovery platform was recently employed by our laboratory in a study that resulted in the unexpected identification of leukemia inhibitory factor (LIF) as a mitogen for BCEC (21). These findings are consistent with earlier studies suggesting the existence of mitogens with a selectivity for tissue-specific EC types (22).

In addition, we found that ADP enhanced angiogenesis in the laser-induced mouse choroidal neovascularization (CNV) model. Interestingly, analysis of single-cell transcriptomics from human choroid datasets (23) shows that P2RY1 is selectively expressed in EC.

## Results

### Identification of ADP as a Mitogen for BCEC.

**Fig. 1.**
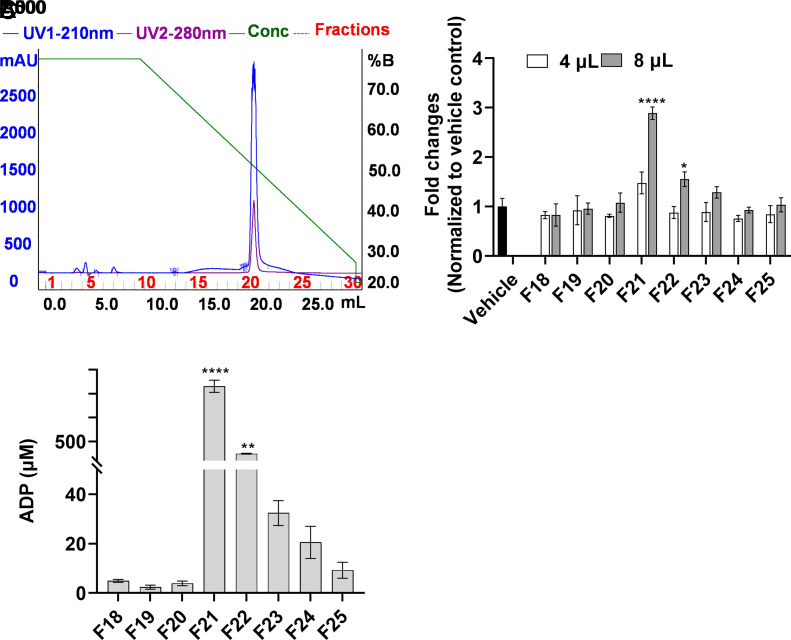
Purification of EC mitogen from EL4 aqueous extracts. Metabolites were extracted from EL4 cells as described in *SI Appendix*, Fig. S1 and were purified through sequential chromatographic steps using HiTrap Q HP 5 mL and HiTrap Q HP 1 mL column (*SI Appendix*, Fig. S3). Active fractions from HiTrapQ 1 mL column were pooled and subjected to HILIC using a TSKgel Amide-80 column, as described in *SI Appendix* (*A*). The y axis represents UV absorbance (mAU) and x axis represents elution volume. The right *y* axis represents % of buffer B. TSKgel Amide-80 column fractions were tested in BCEC proliferation assay (*B*). BCEC plated in 96-well plates were treated with 4 or 8 μL of each fraction, and after 6 d cell proliferation was quantified after adding Cell Viability/Cytotoxicity assay reagent as described in *SI Appendix*. Fraction 21 significantly stimulated the growth of BCEC. Vehicle was used as control to calculate the fold changes in treated samples. Column fractions were dried and reconstituted in water to remove acetonitrile/TFA before testing on BCEC proliferation assay and ADP measurement. The data were analyzed by two-way ANOVA with multiple comparisons and Bonferroni’s post hoc test, *n* = 3. Asterisks indicate significance over vehicle control. Fractions from the TSKgelAmide-80 column were measured for the presence of ADP using a fluorometric ADP measuring kit (*C*). ADP concentrations were measured in each fraction and compared to the inactive F18, *n* = 2. One-way ANOVA followed by multiple comparisons with Bonferroni’s correction was used as statistical test. Experiments were carried out in three independent studies. The results represent the mean ± SD. *P < 0.05, **P < 0.01, ****P < 0.0001.

We sought to test the hypothesis that small molecules from tumor extracts may stimulate growth of cultured EC. An outline of our tumor cell extraction and purification strategies is shown in *SI Appendix*, Fig. S1. We initially found that Amicon 3 kDa centrifugal flow-through (3 kDa-FT) and C18 Sep-Pak flow-through (C18-FT) from KF28, 4T1 and EL4 tumor cell lines stimulated BCEC growth (*SI Appendix*, Fig. S2 *A–C*). Further, 0.4%, and 0.8% of the material processed from a 80 to 90% confluent 15 cm dish induced significant stimulation of BCEC proliferation compared to vehicle. To identify the mitogenic factor(s), we ultimately chose an EL4 cell line because it grows in suspension culture, so it would be easier to generate sufficient amounts of starting material. Several pilot experiments using C18-FT indicated that the small molecule was polar, anionic, and poorly retained in a C18 hydrophobic bed. Based on those observations, we designed suitable purification steps (*SI Appendix*, Fig. S1). A HiTrap Q 5 mL anion-exchange column was used for enrichment (*SI Appendix*, Fig. S3 *A* and *B*) and a HiTrap Q 1 mL column was used for further purification and desalting (*SI Appendix*, Fig. S3 *C* and *D*). The peak mitogenic activity was detected in fraction 24 from the HiTrap Q 5 mL column, which was eluted in the presence of 0.16 M NaCl. Active fractions (F23, F24, F25) were combined and applied to a HiTrap Q 1 mL column. The BCEC mitogenic activity was eluted in the presence of 500 mM trimethylamine (TMA) (*SI Appendix*, Fig. S3 *C* and *D*). Since the mitogenic activity was poorly retained in a reverse phase hydrophobic bed, hydrophilic interaction chromatography (HILIC), a methodology employed in the separation of polar compounds (24), was used as the final step (Fig. 1 *A* and *B*). The peak mitogenic fraction, fraction 21 (F21) from the TSKgel Amide-80 HILIC column, along with fractions 19 (F19) and 24 (F24), was analyzed by electrospray ionization mass spectrometry (ESI–MS). ESI–MS analysis identified unique peaks at m/z 428 and 450 present only in fraction 21 (*SI Appendix*, Fig. S4*A*), corresponding to the [M + H]^+^ and [M + Na]^+^ molecular ion peaks of ADP, respectively. ESI–MS/MS analysis on the m/z 428 peak generated fragmental peaks of m/z 136 (equivalent to the adenine moiety of ADP) and 348 (equivalent to the removal of one phosphate group from ADP) (*SI Appendix*, Fig. S4*B*). High-resolution ESI-MS analysis on fraction 21 further confirmed that it was ADP (*SI Appendix*, Fig. S4*C*). HILIC column fractions with peak mitogenic activity contained the highest ADP concentration as assessed by a commercially available ADP measuring kit (Fig. 1 *B* and *C*), further confirming the identity of the mitogenic molecule as ADP.

**Fig. 2.**
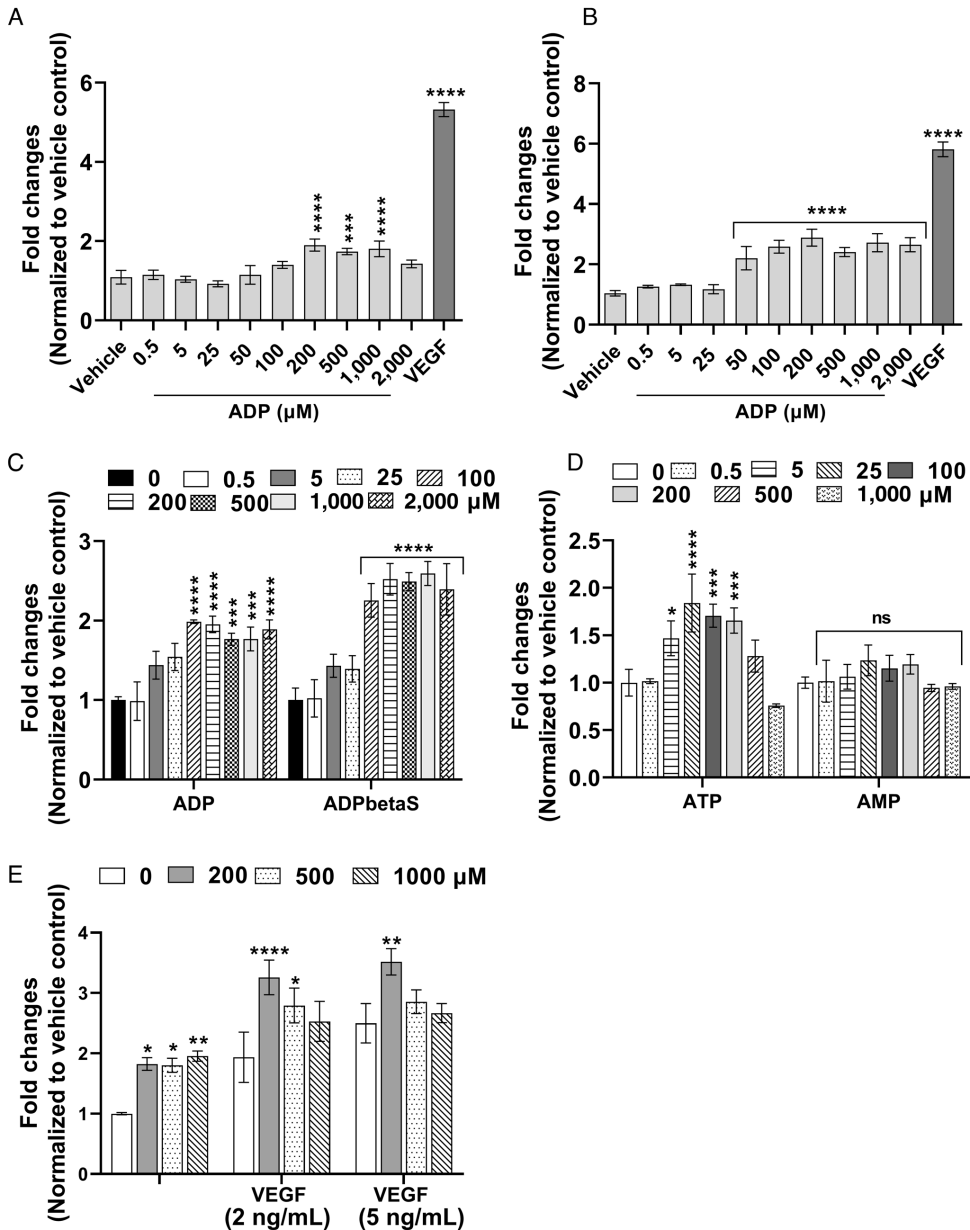
ADP stimulates growth of BCEC and BREC in a dose-dependent manner. BCEC and BREC were cultured in the presence of vehicle, VEGF (10 ng/mL) and the indicated concentrations of ADP and *B*). Vehicle was used as control to calculate fold changes. Effects of ADP were compared to a nonhydrolyzable ADP analog ADP-β-S on BCEC (*C*). Purine nucleotides ATP and AMP were tested on BCEC proliferation assay (*D*). Additive effects of ADP and VEGF on BCEC growth (*E*). BCEC were treated with the indicated concentrations of ADP alone or in the presence of VEGF (2 and 5 ng/mL). Cell proliferation was quantified on day 6, as described in *SI Appendix*. All experiments were carried out in at least three independent studies. Untreated vehicle control cells were used to calculate the fold changes in treated samples. The results represent the mean ± SD, *n* = 3. Asterisks indicate a significant difference compared with vehicle or untreated control in each group. One-way ANOVA and two-way ANOVA followed by multiple comparisons with Bonferroni’s post hoc test was used to calculate the statistical significance. *P < 0.05, **P < 0.01, ***P <0.001, ****P < 0.0001, ns—not statistically significant.

Commercially available ADP stimulated the growth of BCEC and bovine retinal endothelial cells (BREC) at 100 to 1,000 µM, with maximum stimulation observed at 200 µM (Fig. 2 *A* and *B*). A nonhydrolyzable ADP (ADP-β-S) analog also stimulated BCEC growth, and the fold stimulation was higher compared to ADP (Fig. 2*C*). Among other adenine nucleotides, ATP also stimulates BCEC proliferation. However, AMP had no effect (Fig. 2*D*). ADP at 200 and 500 µM showed a significant synergistic effect with 2 ng/mL of VEGF in promoting BCEC proliferation (Fig. 2*E*). We also tested mitogenic effects of ADP on several human microvascular EC. ADP at higher concentrations (500 to 1,000 µM) stimulated proliferation of human dermal microvascular EC (HDMEC), human liver sinusoidal EC (LSEC), human choroidal EC (HCEC), and human retinal microvascular EC (HREC) (*SI Appendix*, Fig. S5).

### Stimulation of BCEC Growth Is Mediated by the P2Y1 Purinergic Receptor and the MAP Kinase Pathway.

**Fig. 3.**
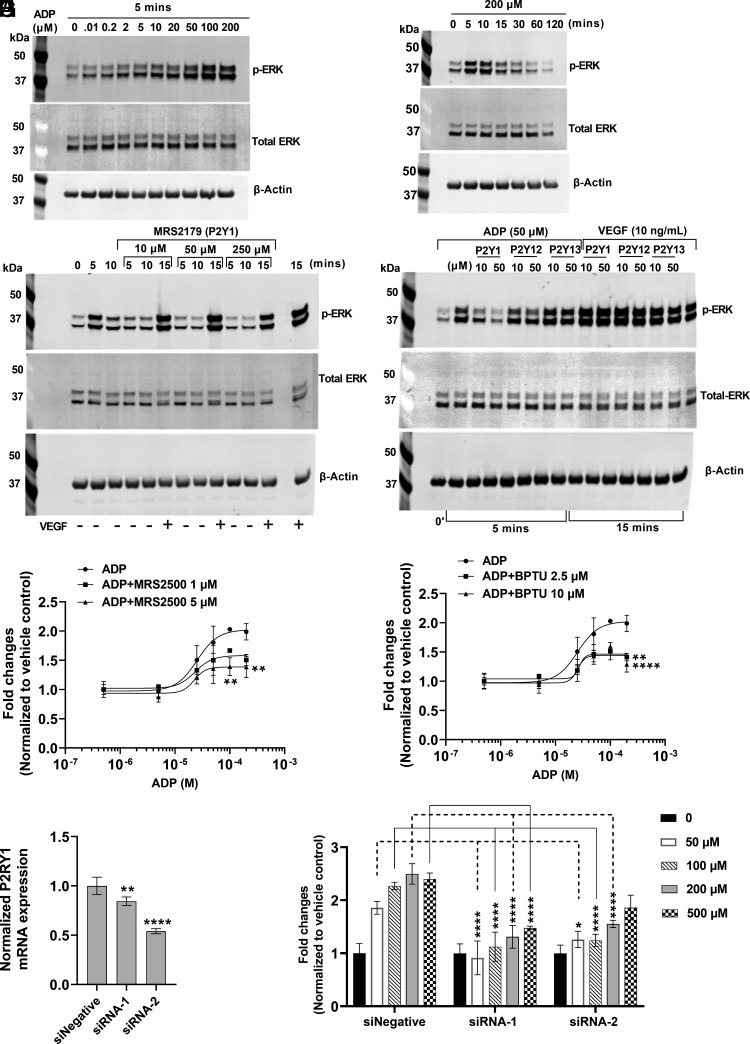
ADP mediates signaling via phosphorylation of ERK1/2 through the P2Y1 purinergic receptor. The cellular phosphorylation levels of different signaling molecules were determined by western blot analysis. BCEC were serum-starved and incubated with increasing concentration of ADP for 5 min for dose–response (*A*) and with 200 µM ADP for time-course (*B*). BCEC were preincubated with 10, 50, and 250 μM of the specific P2Y1 antagonist MRS2179 for 30 min, before stimulation with 50 µM ADP or VEGF (10 ng/mL) (*C*). Cells were harvested 5 and 10 min after ADP addition and after 15 min of VEGF addition. BCEC were preincubated with 10 and 50 µM concentrations of antagonists specific for P2Y1 (MRS 2179), P2Y12 (PSB 0739), and P2Y13 (MRS 2211) for 30 min before stimulating with ADP (50 µM) or VEGF (10 ng/mL) (*D*). Whole-cell lysates were prepared after the indicated time points and subjected to western blotting for phospho-ERK, total ERK and β -actin. Blots were imaged using the LICOR system, which enables simultaneous detection of both total and phosphorylated protein (*SI Appendix*, *SI Materials and Methods*). Since the ERK and β-actin bands have similar molecular weights, stripping would be needed to reprobe the membranes for β-actin. Because of the potential artifacts associated with stripping, we chose to run duplicate gels in parallel in order to perform the β-actin blots. All western blots were done three times, with similar results. BCEC plated in 96-well plates were preincubated with different concentrations of P2Y1 specific antagonists; MRS 2500 (*E*) and BPTU (*F*) for 1 h before stimulating with ADP (0.5, 5, 25, 50, 100, 200 µM). Untreated cells were used as control to calculate the fold changes in treated samples, *n* = 3. Asterisks indicate significant difference over respective ADP alone concentrations. P2RY1 expression was knocked-down by using two different siRNAs in BCEC and knock-down efficiency was quantified by qPCR for P2RY1 using Applied Biosystem Taqman chemistry (*G*). P2RY1 expression in siNegative was considered as control to calculate the fold changes, *n* = 4. Asterisks indicate significance over siNegative. At 24 h post siRNA transfection, cells were plated for proliferation assay in a 96-well plate. Cells were treated with 50, 100, 200, 500 µM of ADP. Cell proliferation was measured at day 5 (*H*). Untreated cells were used as control to calculate the fold changes in treated samples, *n* = 3. A line was drawn to define comparison between specific groups, asterisks indicate significance over respective ADP concentrations in siNegative group. All experiments were carried out in three independent studies. The results represent the mean ± SD. One-way ANOVA and two-way ANOVA followed by multiple comparisons with Bonferroni’s post hoc test was used to calculate the statistical significance. *P < 0.05, **P < 0.01, ****P < 0.0001.

To elucidate the pathways responsible for ADP-induced BCEC growth, cells were treated with increasing concentrations of ADP and key signaling molecules were analyzed at different time points. ADP dose-dependently induced ERK phosphorylation, with maximal effects observed at 100 to 200 µM and within 5 to 10 mins after addition (Fig. 3 *A* and *B*). ADP signaling is mediated by purinergic receptors P2Y, especially through P2Y1, P2Y12, and P2Y13 subtypes (25). To determine which purinergic receptor subtype(s) is responsible for ADP-induced signaling in BCEC, we tested the effects of inhibitors specific for each subtype (25, 26). Preincubation with MRS 2179, specific for P2Y1, completely blocked ADP-induced ERK phosphorylation (Fig. 3*C*). However, antagonists specific for P2Y12 or P2Y13 had no effect (Fig. 3*D*). None of the inhibitors tested blocked VEGF-induced ERK phosphorylation (Fig. 3 *C* and *D*). The role of the P2Y1 receptor was further characterized by pretreating BCEC with P2Y1-specific receptor antagonists MRS 2500 and BPTU before ADP addition (27). Both antagonists resulted in significant inhibition of ADP-stimulated BCEC proliferation at 200 µM concentration (Fig. 3 *E* and *F*). We also tested the effects of ADP on BCEC proliferation after transfection with two different siRNA constructs targeting P2RY1 (Fig. 3*G*). Two independent siRNAs reduced the P2RY1 expression compared to the non-specific negative siRNA control, and downregulation of P2RY1 expression resulted in marked inhibition of ADP-induced BCEC proliferation (Fig. 3 *G* and *H*), indicating the role of P2Y1 receptor in mitogenic effect of ADP.

**Fig. 4.**
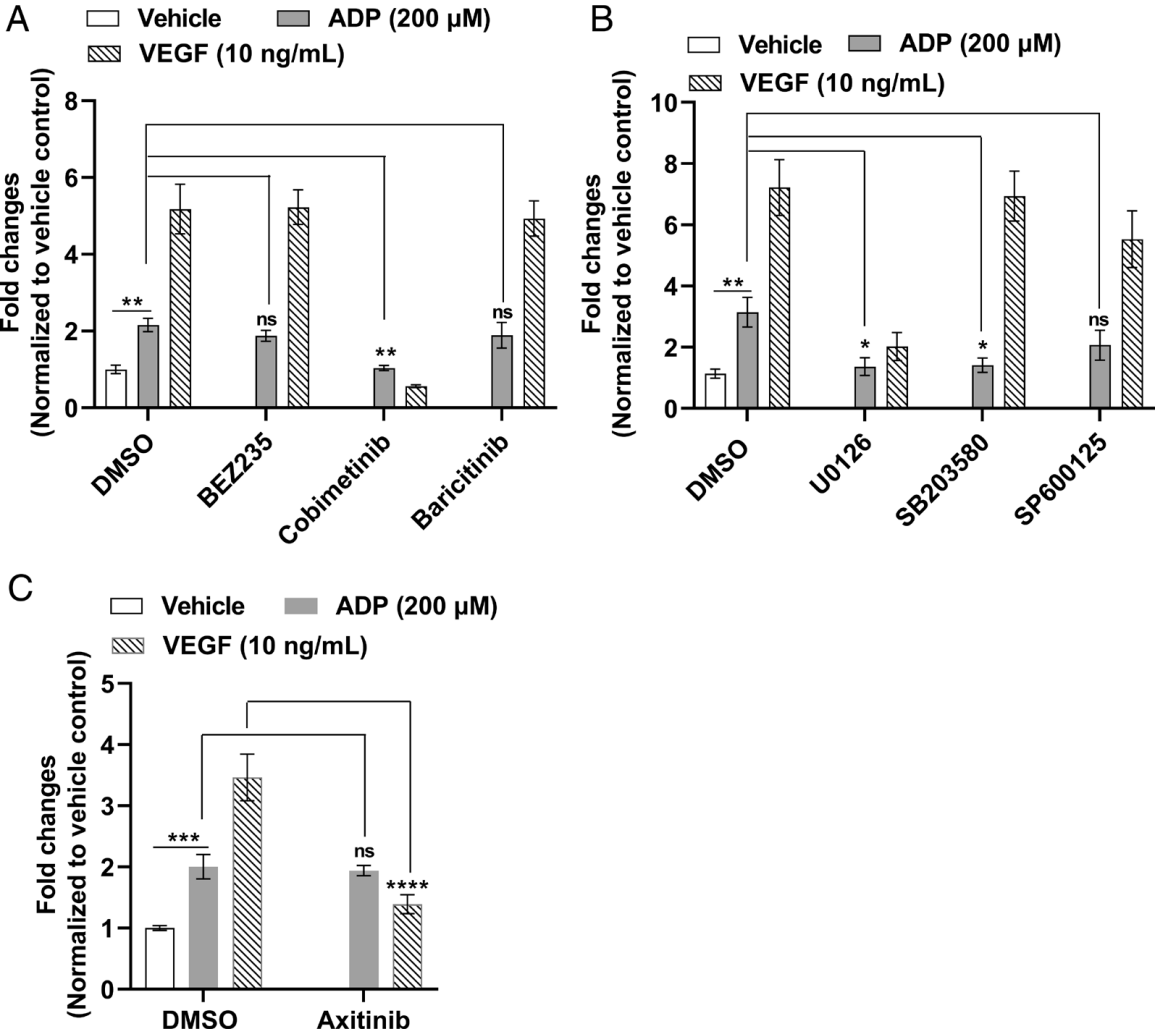
Effects of pharmacological inhibitors on ADP-stimulated BCEC growth. BCEC were plated and treated with BEZ235 (5 nM, specific for PI3K/mTOR pathway), cobimetinib (150 nM, specific for MAPK pathway), and baricitinib (1 µM, specific for JAK/STAT pathway) for 1 h, followed by addition of 200 µM ADP (*A*). To clarify the role of different MAPK kinases, cells were treated with 2.5 µM U0126 (specific for ERK1/ERK2), SB203580 (specific for P38 kinase) and SP600125 (JNK1/2) for 1 h before stimulating with 200 µM ADP (*B*). In (*C*), cells were preincubated with 10 nM axitinib (specific for the VEGF pathway) for 1 h before ADP addition. Cell proliferation was measured by Cell Viability/Cytotoxicity assay reagent on day 6. DMSO-treated samples were used as control to calculate the fold changes in inhibitor treated samples. Data shown are the representative of results from three independent experiments as mean ± SD, *n* = 3. Two-way ANOVA followed by multiple comparisons with Bonferroni’s correction was used to calculate the statistical significance. A line was drawn to define comparison between specific groups. *P < 0.05, **P<0.01, ***P<0.001. ns—not statistically significant.

ADP-induced signaling was further characterized using a set of small-molecule inhibitors specific for MEK1/2 (MAPK, cobimetinib) (28), JAK1/2 (baricitinib) (29), and PI3K/mTOR (BEZ235) (30) pathways. Cobimetinib at 150 nM concentration significantly blocked both ADP and VEGF-induced BCEC proliferation (Fig. 4*A*). To further explore the involvement of the MAPK pathway, we used a set of inhibitors targeting ERK1/2, P38, and JNK1/2 (31). U0126 specific for ERK1/2 significantly blocked both ADP and VEGF-induced cell growth (Fig. 4*B*). The P38 specific inhibitor SB203580 also blocked ADP-induced cell proliferation but had no effect on VEGF-induced cell growth. The JNK inhibitor had no effect on ADP-induced cell growth (Fig. 4*B*). Notably, axitinib, an FDA-approved small-molecule tyrosine kinase inhibitor that potently inhibits the VEGF pathway (32), had no effect on ADP-stimulated BCEC growth, while it completely blocked VEGF-stimulated mitogenesis (Fig. 4*C*). These observations demonstrate the contribution of the MAPK pathway, mainly ERK1/2 and P38, in ADP-stimulated BCEC proliferation and argue against the involvement of the VEGF signaling pathway in mediating ADP-stimulated BCEC proliferation.

### Single-Cell Transcriptomic Analysis of ADP-Specific Purinergic Receptor Expression.

**Fig. 5.**
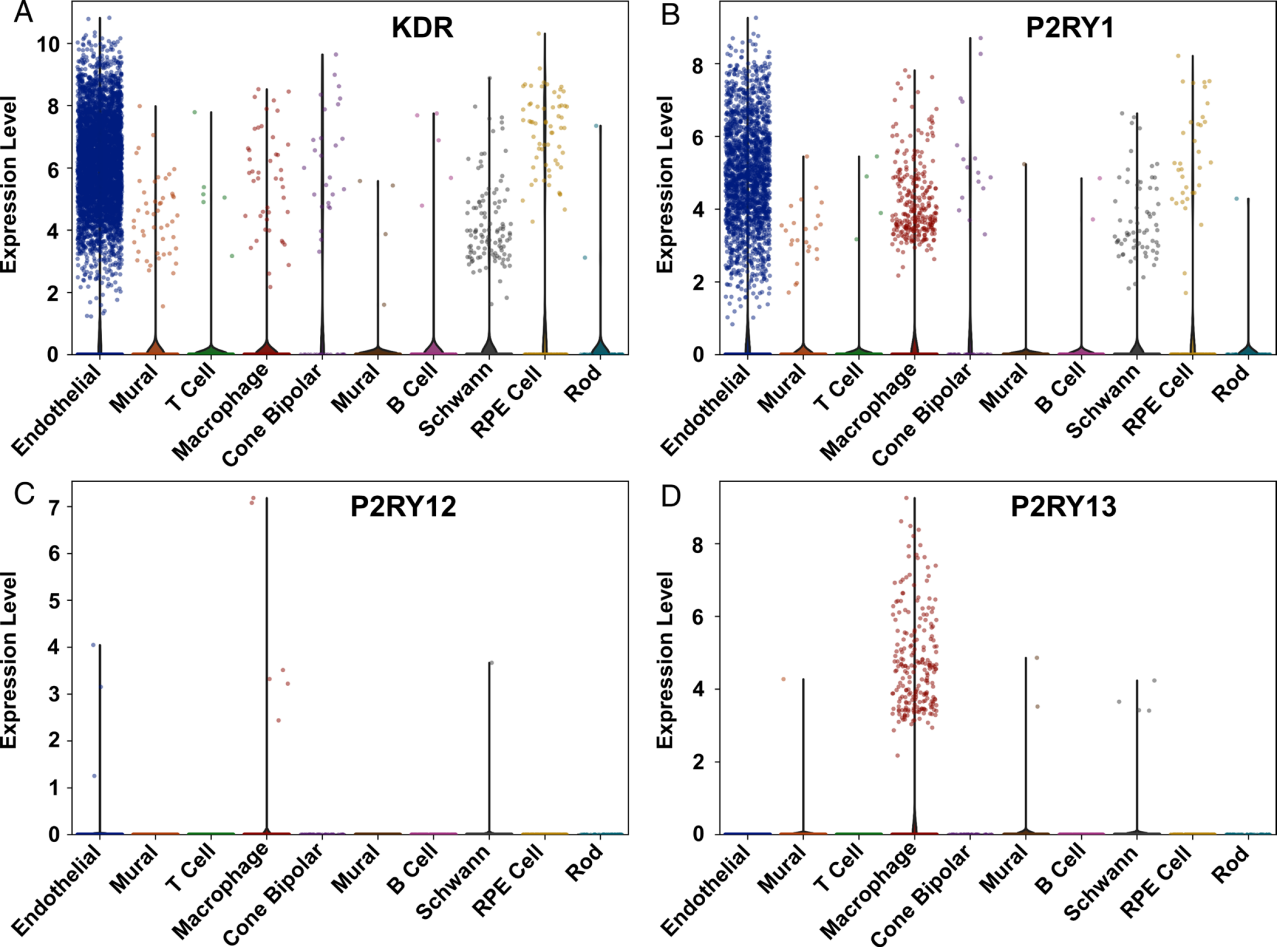
Expression of ADP and VEGF receptors across cell types in the human choroid. Violin plots overlaid with strip plots illustrate the cell-normalized, log2-transformed expression of four receptors: KDR (VEGFR2) (*A*), P2RY1 (*B*), P2RY12 (*C*), and P2RY13 (*D*) across various cell types. Single-cell gene expression data are derived from GSE135922 (23). (See *SI Appendix* *f*or details).

The data were derived from CD31-enriched RPE/choroid cells (23). The plots were obtained using 13,700 cells, of which 7,933 were identified as endothelial cells, representing 58%. A significant portion of endothelial cells (4,515) exhibits nonzero expression of the VEGF receptor KDR (Fig. 5*A*) with an average unique molecular identifier (UMI) count of 4.5. P2RY1 is expressed in 1,915 endothelial cells (Fig. 5*B*), with an average UMI count of 1.9, indicating selective expression in this cell type. The expression of P2RY12 and P2RY13 is sparse, with P2RY13 predominantly expressed in 223 macrophages (Fig. 5 *C* and *D*).

### ADP-Induced Expression of P2RY1 and PlGF in BCEC.

Biochemical characterization of ADP signaling in BCEC pointed to the involvement of a specific P2Y receptor subtype, P2Y1. We therefore examined whether expression of P2RY1 in BCEC is regulated by ADP by real time qPCR. ADP at 200 µM concentration significantly upregulated the P2RY1 messenger RNA (*SI Appendix*, Fig. S6*A*). To further characterize the downstream effects of ADP treatment in BCEC, expression of several growth factors was analyzed by qPCR. ADP increased expression of placental growth factor (PGF/PIGF) which is a member of VEGF subfamily, that acts as a proangiogenic molecule in part through the displacement of VEGF bound to VEGFR1, making it available to bind the signaling receptor VEGFR2 (33) (*SI Appendix*, Fig. S6*B*).

### ADP Stimulates Choroidal Angiogenesis in a Mouse CNV Model.

**Fig. 6.**
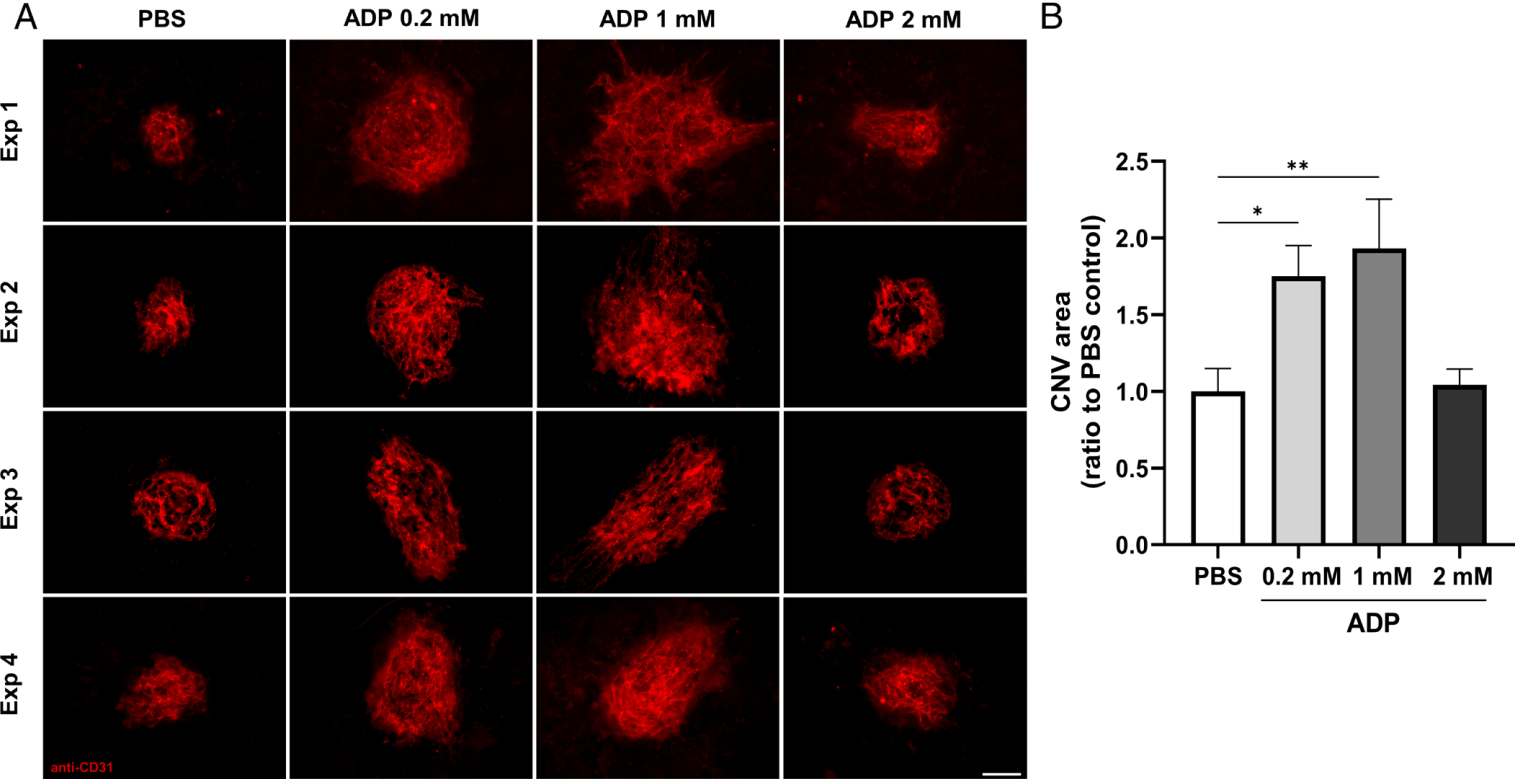
ADP stimulates angiogenesis in a mouse CNV model. (*A*) Immunofluorescence enhanced laser‐induced CNV. Intravitreal administration of ADP (0.2 mM, 1.0 mM and 2.0 mM) after laser‐induced CNV. PBS was used as vehicle control. CD31 immunostaining of choroid flat mounts were carried out 7 d after laser induction, *n* = 5. (Scale bar, 100 µm.) (*B*) Quantification of the CNV area is shown. The column bar graph shows data from four independent experiments. One-way ANOVA followed by multiple comparisons with Bonferroni’s correction was used to calculate statistical significance. The results represent the mean ± SD. *P < 0.05, **P < 0.01.

Laser-induced rupture of Bruch’s membrane is a well-established technique to induce CNV in multiple species (34, 35). To test whether ADP can stimulate choriocapillaris growth, different doses of ADP were injected intravitreally in mice after laser induction. After 7 d, quantification of the lesion areas was performed after flat‐mount CD31 staining. The CNV areas were significantly larger after the 200 µM and 1,000 µM ADP intravitreal injection compared to the PBS control group. However, the 2 mM dose of ADP did not increase CNV area, suggesting a bell-shaped dose response (Fig. 6 *A* and *B*).

## Discussion

In this study, we report the detection of a small molecule mitogen for BCEC in tumor cell aqueous extracts and identification of the mitogenic factor as ADP. Using biochemical and pharmacological approaches, we characterized the mitogenic effects of ADP on BCEC and showed that these are mediated by the P2Y1 subtype receptor in a VEGF-independent manner. Although ADP has been shown to inhibit growth of macrovascular EC, this study demonstrates opposite effects in microvascular EC. Also, intravitreal administration of ADP increased CNV size in a widely used mouse model of neovascular AMD. Strong expression of P2RY1 in human choroidal EC by single-cell transcriptomics analysis is consistent with the hypothesis that ADP may function as a mitogenic and proangiogenic molecule for choroidal EC.

We also examined the possibility that ADP may induce expression of mitogenic factors in BCEC that could modulate the direct effects of ADP. It has been reported that, in response to signals such as VEGF, EC may be instructed to release growth factors (“angiocrine factors”) able to protect tissues from toxic substances and promote regeneration (36, 37). We found that PIGF expression was markedly upregulated by ADP in BCEC. Although the role of PlGF in angiogenesis is complex, it can displace VEGF bound to the poorly signaling VEGFR1, making it available to interact with VEGFR2, thus potentially amplifying the angiogenic cascade [reviewed in (4)].

ADP is stored in the dense granules of platelets and, following secretion, plays a major role in hemostasis by directly stimulating platelet aggregation and also potentiating aggregation responses induced by other agents (38, 39). Nucleotides are ubiquitous extracellular signaling molecules released in extracellular fluids from cell lysis, exocytosis, or efflux through transport proteins. They can function as both paracrine and autocrine molecules, affecting a variety of cellular functions including proliferation, differentiation and death by interacting with plasma membrane P2 purinergic receptors (25). Although ADP is known as a constituent of nucleic acids and for its role in energy metabolism, it also acts as an important signaling molecule that activates cell surface receptors in a broad spectrum of cells. ADP signaling pathways in platelets have been extensively characterized, and ADP receptor P2Y12-specific antagonist clopidogrel is in widespread clinical use (40, 41).

Platelets also play key roles in a variety of pathological conditions, including atherosclerosis and thrombosis, and have been also implicated in tumor growth, chemoresistance, and metastasis (42). Increased release of ATP, ADP, and their metabolites from dying cells plays a role in tumor angiogenesis and cancer-associated thrombosis (43). Identification of ADP as an EC mitogen could be relevant in the context of interaction between platelets and areas of neovascularization such as those occurring in tumors and intraocular diseases. Platelets have been shown to colocalize next to the damaged EC in diabetic retinal vasculature and to reduce blood retinal barrier breakdown (44). Growth stimulation of BCEC in the presence of 200 µM ADP is likely physiologically significant since the concentrations of ATP and ADP in platelet dense granules are reported to be very high (45, 46). Thus, ADP released upon platelet activation can reach levels that can stimulate the proliferation of nearby EC. However, the role of platelets in retinal neovascularization is complex and, at least in the context of the retinopathy of prematurity, low platelet counts have been associated with increased neovascularization (47), which has been attributed to reduced VEGF scavenger/storage functions mediated by platelets (48).

A variety of published studies have yielded multiple and at times conflicting results on the effect of nucleotides on EC proliferation. ADP has been shown to promote HUVEC proliferation at very low concentrations, inhibit at high concentrations, and promote migration via P2Y1 receptor (20, 46). Also, ADP has been reported to inhibit the proliferation of BAEC and HAEC (49). The function of ADP on various EC might depend on the type of P2 receptors expressed, the balance of release and degradation of nucleotides, as well as the presence of another messenger. Nucleoside triphosphate diphosphohydrolase (NTP Dase or ecto ATPase CD39) and ecto 5’-nucleotidase/CD73 are major enzymes responsible in converting ATP to ADP, then AMP, and finally adenosine, and thus regulate the duration and magnitude of purinergic signaling. In our hands, among adenine nucleotides, ATP and ADP showed BCEC stimulatory properties, whereas AMP was ineffective. Adenosine has been shown to mediate hypoxic induction of VEGF in retinal EC and pericytes (50) and to stimulate human retinal EC proliferation, which could be blocked by an anti-VEGF antibody (51). However, in contrast to the observations with adenosine, blocking the VEGF pathway with axitinib had no effect on ADP-induced BCEC proliferation, consistent with direct EC mitogenic effects of ADP.

VEGF inhibitors have had a dramatic impact on the treatment of retinal and CNV (5). However, not all patients respond and thus there still is an unmet medical need. It is conceivable that EC damage and hemorrhage and the resulting formation of microthrombi may lead to the release of, among other metabolites, ADP, which can promote VEGF-independent angiogenesis. A better understanding of the modulation of specific purinergic receptors P2X versus P2Y in the RPE and retina will be needed to clearly establish the role of ADP in AMD pathogenesis. *SI Appendix*, Fig. S7 depicts a model of the potential role of ADP vs. VEGF in inducing choroidal angiogenesis.

Numerous metabolites exist in cell extracts; some are nonpolar or unstable and are likely lost during purification. Modified strategies targeted for other organic molecules and employing other organ-specific EC types will be helpful to identify novel bioactive metabolites in future studies. Our results, along with P2RY1 expression in HCEC, may have implications for translational research: by delivering P2Y1 selective stable agonist to promote angiogenesis after injury or by blocking P2Y1 to control abnormal angiogenesis.

## Materials and Methods

EL4 cells were grown in high glucose DMEM with 10% FBS and antibiotic in T175 flasks in several batches and subjected to metabolites extraction. Fractions from each purification step were tested for the presence of mitogenic activity on BCEC proliferation assay as described (35). Detailed procedures are described in *SI Appendix*. Animal studies were performed in compliance with University of California, San Diego Institutional Animal Care and Use Committee.

## Supplementary Material

Appendix 01 (PDF)

## Data Availability

All study data are included in the article and/or *SI Appendix*.
