# Supplementary material for: Adenosine diphosphate stimulates VEGF-independent choroidal endothelial cell proliferation: A potential escape from anti-VEGF therapy
Source: Proc Natl Acad Sci U S A. 2025 Jan 21;122(4):e2418752122. doi: 10.1073/pnas.2418752122 (PMC11789014; doi:10.1073/pnas.2418752122)
Supplement: Supplementary file 1 — Appendix 01 (PDF) [file pnas.2418752122.sapp.pdf]

Supporting Information for

Adenosine Diphosphate Stimulates VEGF-Independent Choroidal Endothelial Cell Proliferation: A Potential Escape from anti-VEGF Therapy

Nilima Biswas[1], Tommaso Mori[1], Naresh Kumar Ragava Chetty Nagaraj[1], Hong Xin[1], Tanja Diemer[1], Pin Li[1], Yongxuan Su[3], Carlo Piermarocchi[4], Napoleone Ferrara[1,2*]

[1] Department of Pathology, University of California San Diego, La Jolla, CA, 92093

[2] Department of Ophthalmology, University of California San Diego, La Jolla, CA, 92093

[3] Department of Chemistry and Biochemistry, University of California San Diego, La Jolla, CA, 92093

[4] Department of Physics and Astronomy, Michigan State University, East Lansing, MI, 48824

T.M. and N.K.R.C.N. are equal contributors

*Corresponding author**:** Napoleone Ferrara

Email: nferrara@ucsd.edu

This PDF file includes:

SI Materials and Methods

Figures S1- S7

SI References

Supplementary Information Text

SI Materials and Methods

Chemicals. Adenosine 5'-diphosphate sodium salt (ADP) (Cat # A2754), adenosine 5'-triphosphate disodium salt hydrate (ATP) (Cat # A7699), adenosine 5'-monophosphate sodium salt (AMP) (Cat # A1752), adenosine 5'-[β-thio]-diphosphate trilithium salt (ADP-β-S) (Cat # A8016) were obtained from Millipore Sigma. Small molecule inhibitors: BEZ235 (Cat # S1009), baricitinib (Cat # S2851), cobimetinib (Cat # S8041), SB203580 (Cat # S1076), SP600125 (Cat # S1460) were purchased from Selleck Chemicals. U0126 (Cat # 9903) and axitinib (Cat # 12961) were from Cell Signaling Technology. MRS 2179 tetrasodium salt (Cat # 0900), MRS 2211 (Cat # 2402), PSB 0739 (Cat # 3983), BPTU (Cat # 6078) and MRS 2500 tetraammonium salt (Cat # 2159) were from TOCRIS Bioscience. Trimethylamine aqueous solution 40% (Cat # 8.21177) was from Millipore Sigma.

Antibodies. Anti-total ERK1/2 (Cat # 4696) and anti-phospho ERK1/2 (Thr 202/Tyr 204, Cat # 4370) were from Cell Signaling Inc. Anti-β-actin antibody was purchased from Sigma (Cat # A1978).

Cell cultures. Mouse lymphoma EL4 (TIB-39™, ATCC) cells were maintained in Dulbecco's Modified Eagle Medium (DMEM) high-glucose supplemented with 10% FBS; KF28 cells were maintained in RPMI with 10% FBS; 4T1 cells were maintained in DMEM high-glucose with 10% FBS. Bovine choroidal endothelial cells (BCEC) (VEC Technologies, Rensselaer, NY, Cat # BCME-4) (Passage 5-9) and bovine retinal endothelial cells (BREC) (VEC Technologies, Rensselaer, NY, Cat # BRME-3) (Passage 5-9) were maintained in low-glucose DMEM

supplemented with 10% bovine calf serum (BCS), 2 mM glutamine, 5 ng/mL bFGF, and 10 ng/mL human $VEGF_{165}$ in fibronectin (or 0.1% gelatin coated) culture plates. Human choroidal endothelial cells (HCEC) (Celprogen, Torrance, CA, Cat # 36052-03) were maintained in choroidal endothelial growth medium (Celprogen, Cat # M36052-03S) with antibiotics in gelatin-coated culture plates. Human retinal microvascular endothelial cells (HREC) (Cell Systems, Cat # ACBRI 181), human dermal microvascular endothelial cells (HDMEC) (Lonza, Cat # CC-2543), and human liver sinusoidal microvascular endothelial cells (LSEC) (Cell Systems, Cat # ACBRI 566) were cultured in EGM-2 MV medium (Lonza, Cat # CC-3202). Cells were maintained at 37°C in a humidified atmosphere with 5% CO_2. $VEGF_{165}$ (Cat # 293-VE) and bFGF (Cat # 233-FB) were purchased from R & D systems.

Endothelial cell (EC) proliferation assays. BCEC proliferation assays were performed essentially as described previously (1-3). To assess the proliferative effects of test compounds, BCEC or BREC were seeded (800-1000 cells/well in 0.2 mL media) in 96 well plates in DMEM low-glucose supplemented with 2.5% BCS, 2 mM glutamine and antibiotics. Human EC were seeded at a density of $1x10^3$ cells/well in gelatin-coated 96 well plate in serum-free endothelial basal medium (EBM-2, Lonza, Cat # CC-3156) with antibiotics. Recombinant human VEGF was used as a positive control at 10 ng/mL. In studies employing small molecule inhibitors, these were added 1 hr prior to ADP addition. After treatment, cells were incubated for 5-6 days and cell proliferation was determined by Cell Viability/Cytotoxicity assay reagent (Cat # K020, Advanced BioReagents), according to the manufacturer's instructions. Fluorescence was measured at 530 nm excitation and 590 nm emission wavelength using SpectraMax M5, Molecular devices, USA.

Purification of EL4 derived EC mitogen. EL4 cells were cultured in T175 tissue culture flasks in 50 mL medium per flask until they reached a density of $2.5x10^6$ cells/mL. Cells were then

harvested, washed in phosphate buffered saline (PBS), and pellets were frozen and stored at -80° C. Pellets from approximately 500 flasks were combined and processed for extraction using methanol (-80°C), chloroform (-20°C) and water (4°C) at 1:1:0.5 ratio. The combination of solvents for extraction was optimized based on previous reports (4-6). After centrifugation and phase separation, the upper aqueous layer was collected, dried, and reconstituted in water. The aqueous extract was then applied into 15 mL 3 kDa Amicon centrifugal filters (Amicon® Ultra-15, Ultracel-3k) and centrifuged at 4000 g for 55 min. 3 kDa flow-through (3 kDa-FT) was collected, adjusted to 0.1% formic acid (FA), and then applied to a Sep-Pak Vac 20cc (5 gm) C18 cartridge (Waters Corporations, USA) pre-equilibrated in 0.1% FA in water. Flow-through and wash fractions were combined (C18-FT), the pH was raised to 8.0, then diluted in HiTrap Q buffer A (10 mM Tris, pH 8.0). Subsequent chromatographic steps were performed in an AKTA Explorer system. Samples were applied on HiTrap Q HP 5 mL column (Cytiva) equilibrated in buffer A. After application, the column was washed in buffer A and then eluted with a gradient of 0 to 50% buffer B (buffer A with 0.5 M NaCl) in 20 column volumes (CV), followed by 100% buffer B in 10 CV. 2.5 mL fractions were collected and tested on BCEC proliferation. Active fractions were combined, diluted in HiTrap Q buffer A, and re-applied to HiTrap Q HP 1 mL column. The column was washed extensively with buffer A, then sequentially washed with 10 mL of 25-, 250- and 500- mM trimethylamine (TMA), pH 5.0. TMA is volatile and thus suitable for hydrophilic interaction chromatography (HILIC) and mass spectrometry (MS) applications (7). HILIC is a technique employed in the separation of polar compounds (8, 9). 1 mL fractions were collected, dried, washed and reconstituted in water before testing for BCEC proliferation. Active fractions from the 1 mL HiTrap Q column, eluted in 500 mM TMA, were adjusted to 0.1% trifluoroacetic acid (TFA) in 75% acetonitrile and applied to a TSKgel Amide-80 (0.46x25 cm, 5 µm, TOSOH Bioscience LLC)

HILIC column equilibrated in 0.1% TFA in 75% acetonitrile. The column was washed with equilibration buffer and bound compounds were eluted with a gradient of 75% to 25% acetonitrile (0.1% TFA) in 5 CV. 1 mL fractions were collected, dried, washed, reconstituted in water and tested in BCEC proliferation assays.

Mass spectrometry analysis. Mass spectrometry (MS) analysis was performed in the Molecular MS Facility (MMSF) at UC San Diego. A Thermo LCQdeca mass spectrometer was employed for low resolution electrospray ionization mass spectrometry (LR-ESI-MS) and MS/MS analysis (10). The ESI source was operated under positive ion mode with the following parameters: source voltage: 5 kV; sheath gas flow rate: 80 units; auxiliary gas flow rate: 20 units; capillary temperature: 250ºC. XCalibur software (version 2.0) was used for data acquisition and analysis. An Agilent 6230 time-of-flight mass spectrometer (TOFMS) with Jet Stream ESI source was used for high resolution MS (HR-MS) analysis. The Jet Stream ESI source was operated under positive ion mode with the following parameters: VCap: 3500V; fragmentor voltage: 160 V; nozzle voltage: 500 V; drying gas temperature: 325ºC, sheath gas temperature: 325ºC, drying gas flow rate: 7.0 L/min; sheath gas flow rate: 10 L/min, and nebulizer pressure: 40 psi. Agilent MassHunter workstation software (version 10.1) was used for data acquisition and analysis.

ADP measurement. ADP concentrations were measured by a fluorometric kit according to the manufacturer's instruction (Cat # LS-K202-100, LSBio LifeSpan Biosciences, Inc.).

SDS-PAGE and Western blots. BCEC were cultured overnight in complete growth medium and then were switched to growth factor-free media with 1% BCS. Cells were serum-starved for 4-5 hours, followed by treatment with ADP or other small molecules. Finally, cells were washed with cold PBS twice and lysed in RIPA buffer (Cat # 89901, Life Technologies) with protease and phosphatase inhibitor cocktail (Thermo Fisher Scientific, Cat # 78440). Equal amounts of protein were subjected to SDSPAGE, transferred to PVDF membrane,

blocked with blocking buffer and then incubated overnight with primary antibodies. The next day, the membranes were washed with Tris buffered saline – tween 20 (TBST) thrice and incubated with fluorescent secondary antibodies, goat anti-mouse (IRDye 800 CW) and goat-anti rabbit (IRDye 680RD) for 1 hr. After incubation with secondary antibodies, the blots were washed with TBST thrice. The blots were imaged using the LICOR system and Odyssey Imagers (LICORbio, Nebraska), which enable simultaneous detection of both total and phosphorylated proteins. Because the ERK and β-actin bands have similar molecular weights, stripping would be needed to re-probe the membranes for β-actin. Because stripping is known to create artifacts, duplicate gels were run in parallel in order to perform the β-actin blots. Each blot was done three times, with similar results.

Single-cell transcriptomic analysis. Single-cell gene expression data are available from GSE135922 (11). We restricted the analysis to cells obtained from CD31-enriched datasets (donors 4-7) from both the macula and the periphery. The data were processed using pipelines in the Digital Cell Sorter (DCS) software (12, 13) where mRNA counts are first normalized by cell and then log2 transformed. All zero counts are replaced with the smallest normalized value across the entire dataset before log transformation, and only genes with sufficiently high variation across the entire dataset are retained. After quality control and clustering, annotation was performed using a marker-based approach, where we utilized a set of known markers and the annotation algorithm as described (12) to identify the cell types.

SiRNA knockdown. BCEC ($1.5x10^5$/well) were plated in fibronectin coated 6-well culture plates overnight in complete growth media. The next morning, media were replaced with 2 ml of fresh antibiotic-free media before transfection. siRNAs against P2RY1 (Silencer Select pre-designed siRNA n292604 and s9962) and negative control siRNA (Silencer™select negative control

siRNA, 4390843) from Thermo Fisher Scientific were mixed with Lipofectamine RNAiMAX reagent (Thermo Fisher Scientific, Cat # 13778-150) in Opti-MEM™ 1 reduced serum medium (Gibco, Cat # 31985062) according to manufacturer's instruction. Cells were incubated with siRNAs for 8 hr, fresh media was then added. Twenty-four hours after transfection, cells were trypsinized and plated for the proliferation assay; the remaining cells were harvested for RNA extraction.

RNA extraction and qRT-PCR. BCEC were lysed with Trizol reagent (Invitrogen, Cat # 15596026) and RNA was extracted following manufacturer's instructions. High-capacity cDNA reverse transcription kit (Applied Biosystems, Cat # 4368814) was used to reverse transcribe one microgram of total RNA. Equal amounts (10 ng/reaction) of cDNAs were subjected to qRT-PCR using TaqMan Fast Advanced Master Mix (Applied Biosystems, Cat # 4444557) and ViiA7 Real-time PCR system. mRNA levels were normalized to internal control RLPL0. The following TaqMan gene expression assay probes were used: bovine RLPL0 (Bt03218086_m1), bovine P2RY1 (Bt03214185-s1), bovine ANGPT1 (Bt03249559-m1), bovine PGF (Bt03222871-m1), bovine HGF (Bt03220662-m1), bovine FGF2 (Bt032559205-m1), bovine VEGFA (Bt03213282-m1).

Laser-induced choroidal neovascularization (CNV). C57BL/6J mice (6–8 weeks) were anesthetized with a 100mg/Kg Ketamine-20 mg/ Kg Xylazine cocktail before laser treatment. Before experiments, mice were randomly and blindly assigned to different groups. CNV lesions were induced by laser photocoagulation using a diode laser (IRIDEX, Oculight GL) and a slit lamp (Zeiss) with a spot size of 50 µm, power of 180 mW, and exposure duration of 100 ms (14). Four laser burns were typically induced at 3, 6, 9, and 12 o'clock positions around the optic disc in each eye. The indicated concentrations of ADP and PBS vehicle control were injected intravitreally, in a 1 µL volume, with a 33-gauge Hamilton syringe. Seven days after laser

induction, choroid-sclera complexes and retinas were separated and anti-CD31 immunofluorescence (IF) was performed to evidence the vasculature by whole-mount staining of both retina and choroidal tissues. For CD31 IF, rat anti-mouse antibody (Cat # 550274, BD Biosciences) was diluted 1:100 and incubated overnight at 4°C. After 4 h incubation with a secondary anti-rat antibody (Cat # A21434, Invitrogen), whole mounts were imaged at 555 nm. Quantification of neovascularization in lesion area and vascular density in the retina was carried out by Image J.

Statistical analyses. All experiments were carried out in three independent studies, except for the mass spectrometry analyses, and a representative result is shown. Bars and error bars in the figures represent the mean ± standard deviation (SD). All statistical analyses were performed using GraphPad Prism software, version 9 using one-way or two-way ANOVA as appropriate, followed by multiple comparisons along with Bonferroni's post- hoc test. For comparison between the only two groups in a study unpaired *t*-test with Welch's correction was performed. Specific statistical method and the *n* values are mentioned in the figure legends. Significant *P* values are represented in the figures as $^{*}P<0.05$, $^{**}P<0.01$, $^{***}P<0.001$, $^{****}P<0.0001$.

Supplementary Figures

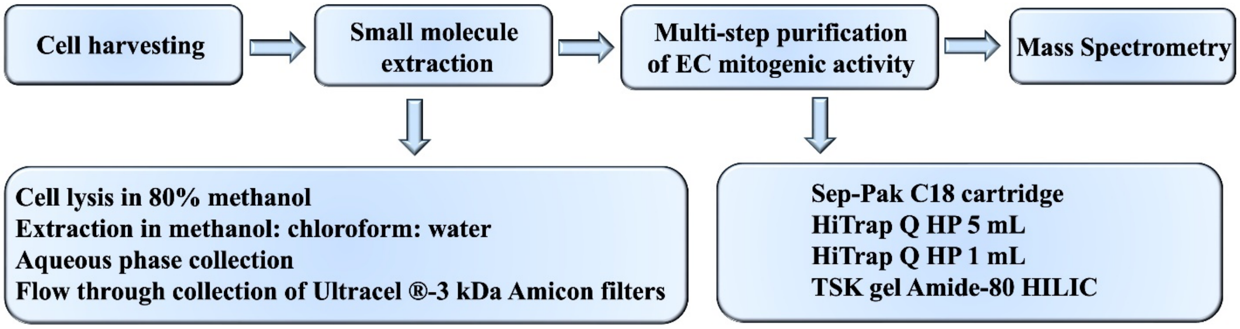

Figure S1. Outline of procedures employed in metabolite extraction and EC mitogen purification. Extraction was performed by brief sonication of tumor cell cell pellets in cold 80% methanol, followed by removal of non-polar metabolites by phase separation. Aqueous extracts were applied to Amicon 3 kDa molecular weight cut-off filters and the flow-through (FT) was collected. FT was acidified and passed through a C18 Sep Pak cartridge. EC mitogenic activity in the C18 FT was purified by three chromatographic steps and finally analyzed by mass spectrometry.

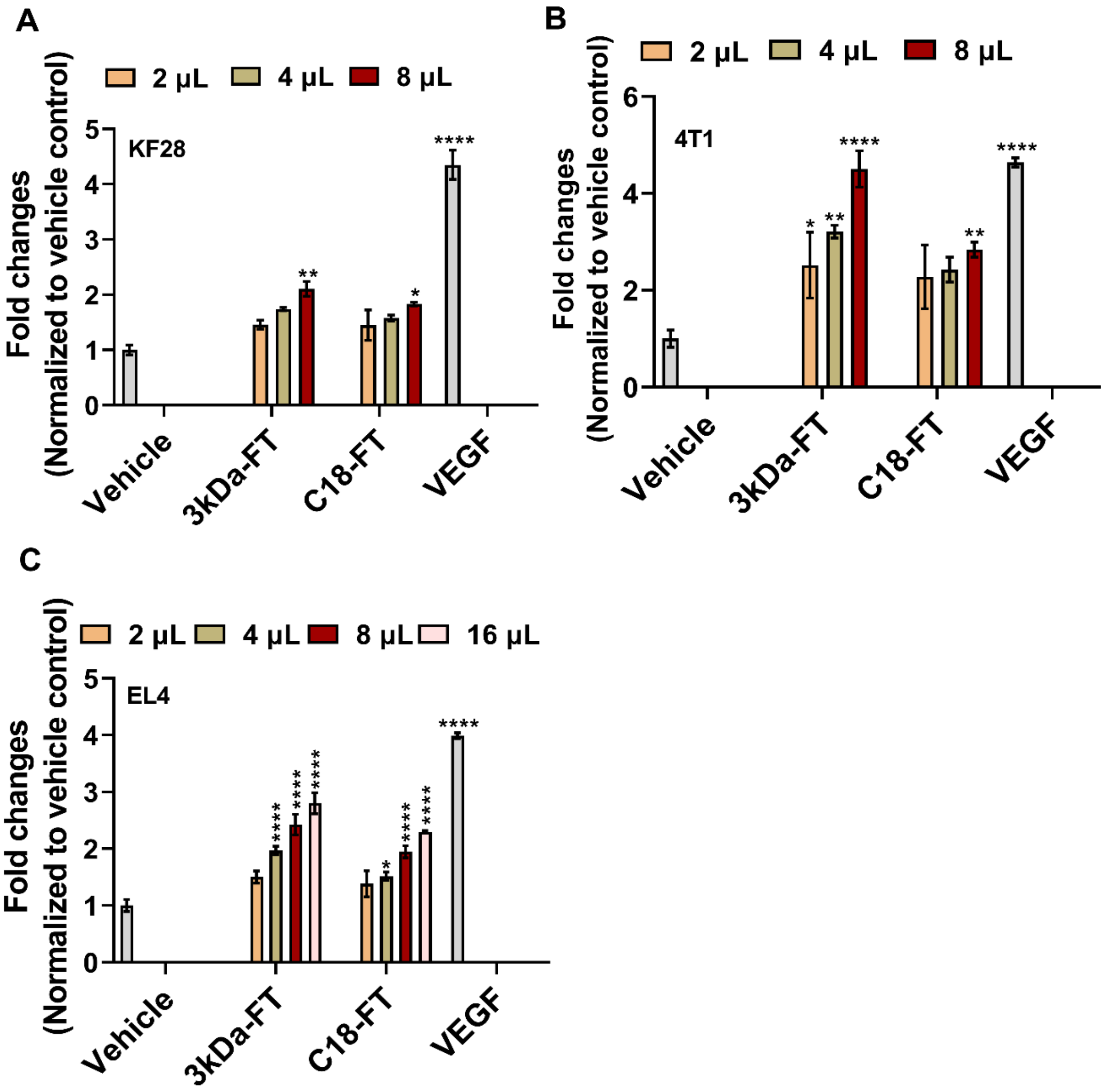

Figure S2. Identification of EC mitogenic activity in aqueous extracts from tumor cell lines (*A*) KF28, (*B*) 4T1 and (*C*) EL4. 3kDa-FT and C18-FT stimulated BCEC growth in a concentration-dependent manner. VEGF (10 ng/mL) was used as positive control. Cell densities were measured at day 6 as described. Vehicle was used as control to calculate the fold changes in treated samples. A representative experiment is shown from three independent studies. The results are presented as the mean ± SD, n = 2. Two-way ANOVA followed by multiple comparisons with Bonferroni's correction was used as statistical test. Asterisks indicate significance over vehicle control. *P<0.05, ** P<0.01, ****P<0.0001.

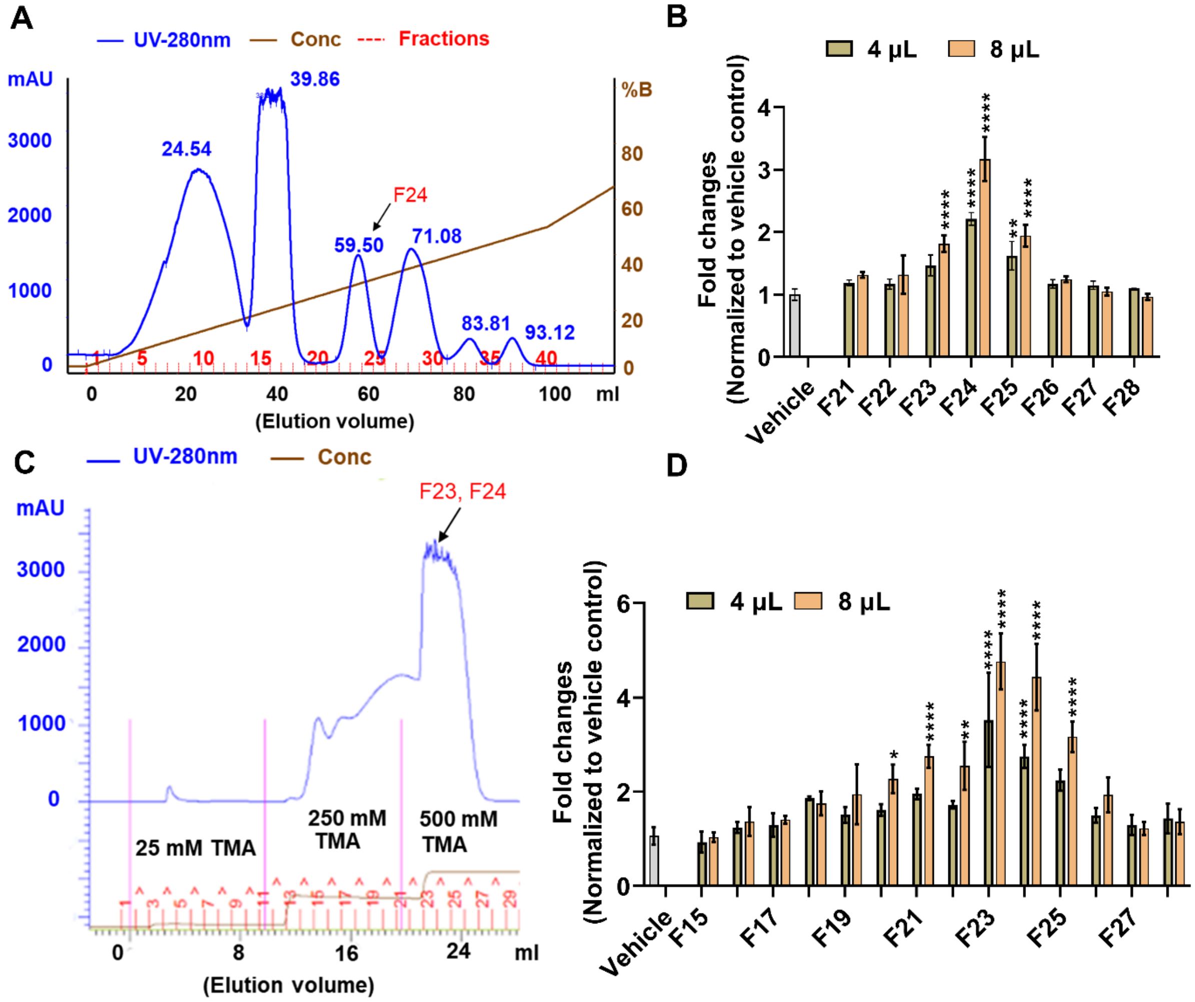

Figure S3. Purification of EC mitogen by ion exchange chromatography.

(***A***) After Amicon 3 kDa filtration and C18 Sep-Pak step, the EL4 cell flow-through was subjected to anion exchange chromatography using HiTrap Q HP 5 mL column. Elution was with a linear, 0 to 0.25 M NaCl gradient, in 20 CV. Left y axis represents absorbance (A_{280}), right y axis represents % of buffer B and x axis represents elution volume in mL.

(***B***) Column fractions 21-28 (F21-F28) from HiTrap Q column were assayed for proliferative effects on BCEC. Cells were plated in 96-well plates and treated with 4 or 8 µL of each fraction. After six days, cell proliferation was quantified. Vehicle was used as control to calculate the fold

changes in treated fractions. Further, fractions having mitogenic activity (F23, F24, F25) were pooled, diluted, and subjected to desalting and concentration using HiTrap Q HP 1 mL column.

(C) Step-wise elution from HiTrap Q 1 mL column was performed with 25-, 250- and 500- mM trimethylamine (TMA) and fractions were collected. Y axis represents A_{280} (mAU), x axis represents elution volume in mL. The TMA was removed from fractions using a speed vac and multiple washes with water and then reconstituted in water.

(D) Fractions from the step-wise elution of HiTrap Q 1 mL column were analyzed for proliferative activity on BCEC. Cell proliferation was measured on day 6. Vehicle was used as control to calculate the fold changes in treated fractions.

A representative experiment is shown from three independent studies. The results are presented as the mean ± SD, $n = 3$. Two-way ANOVA followed by multiple comparisons with Bonferroni's correction was used as statistical test. Asterisks indicate significance over vehicle control. *$P<0.05$, **$P<0.01$, ****$P<0.0001$.

A

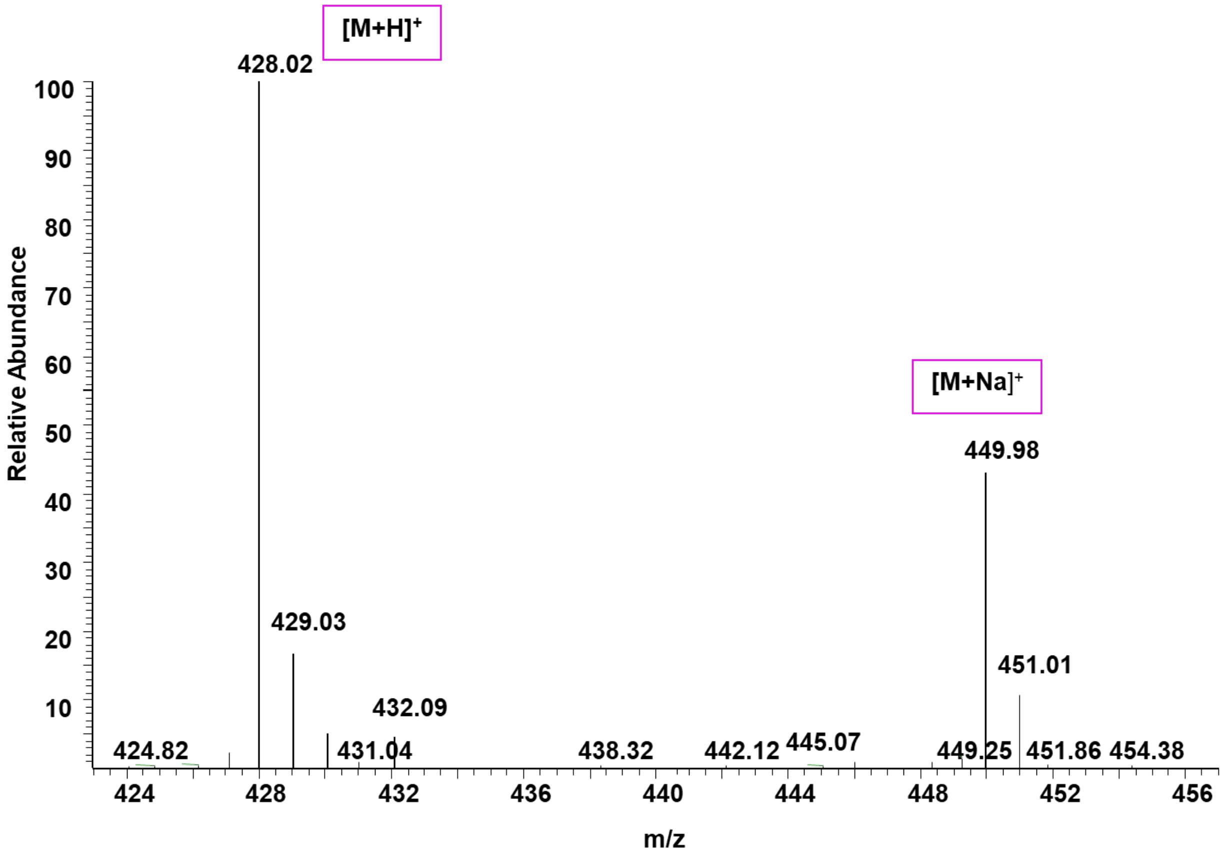

Figure S4-*A*. Mass spectrum of fraction 21 (F21) from TSKgel Amide-80 column. Full-scan ESI-MS spectrum shows the $[M+H]^+$ and $[M+Na]^+$ molecular ion peaks at m/z 428 and m/z 450, respectively.

B

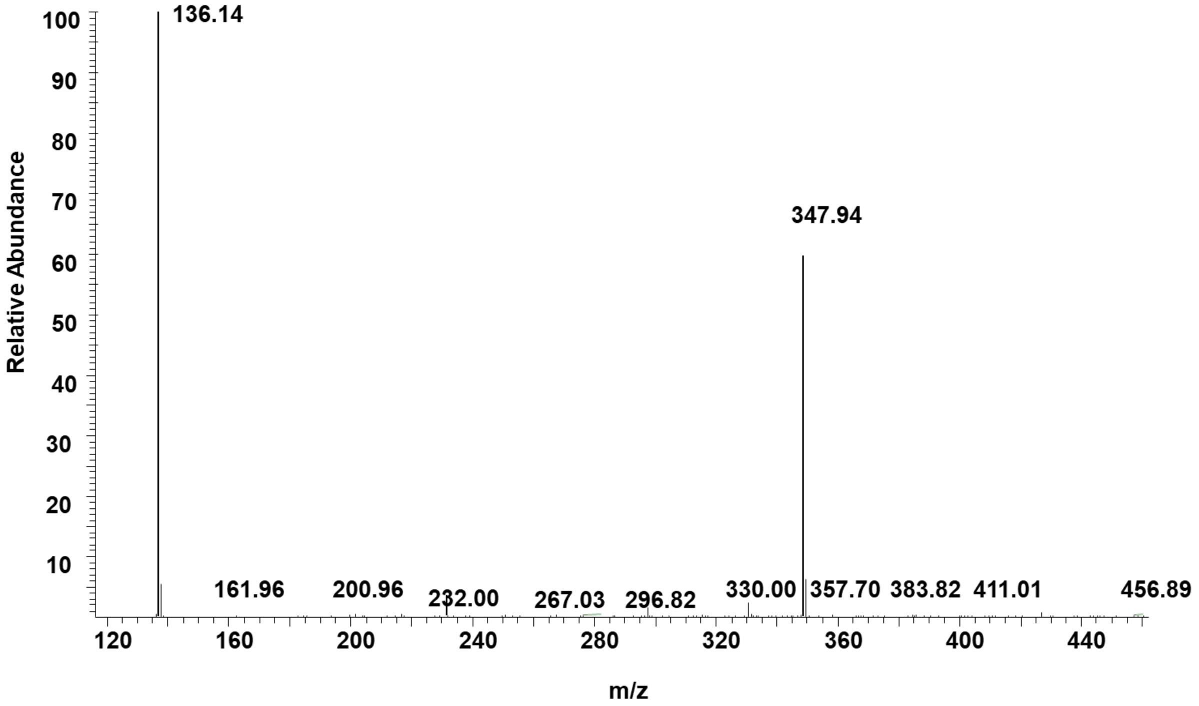

Figure S4-*B*. Mass spectrum of ESI-MS/MS analysis on the $[M+H]^+$ molecular ion peak (m/z 428, as shown in Figure S4-*A*) generated two major fragments at m/z 136 and m/z 348, respectively.

C

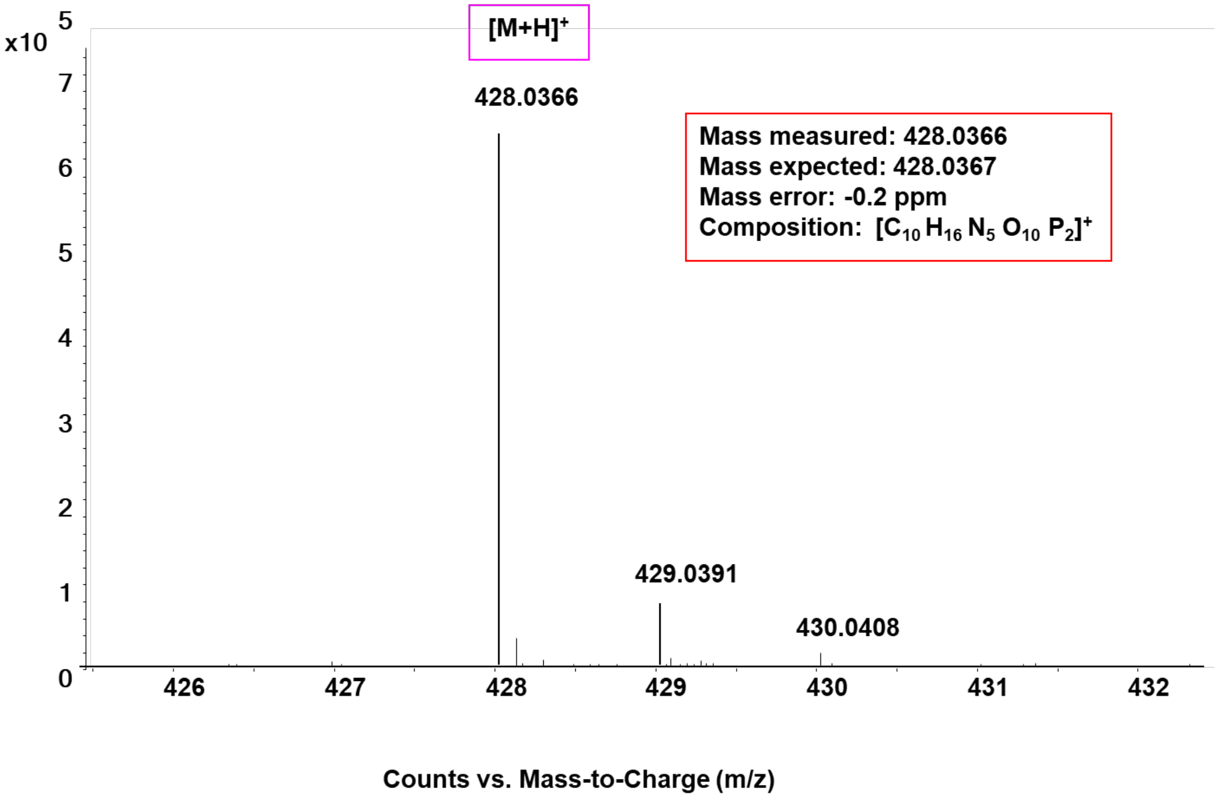

Figure S4-*C*. Mass spectrum of HR-MS measurement confirmed that the m/z 428 peak has the same elemental composition as ADP (Adenosine 5'-diphosphate).

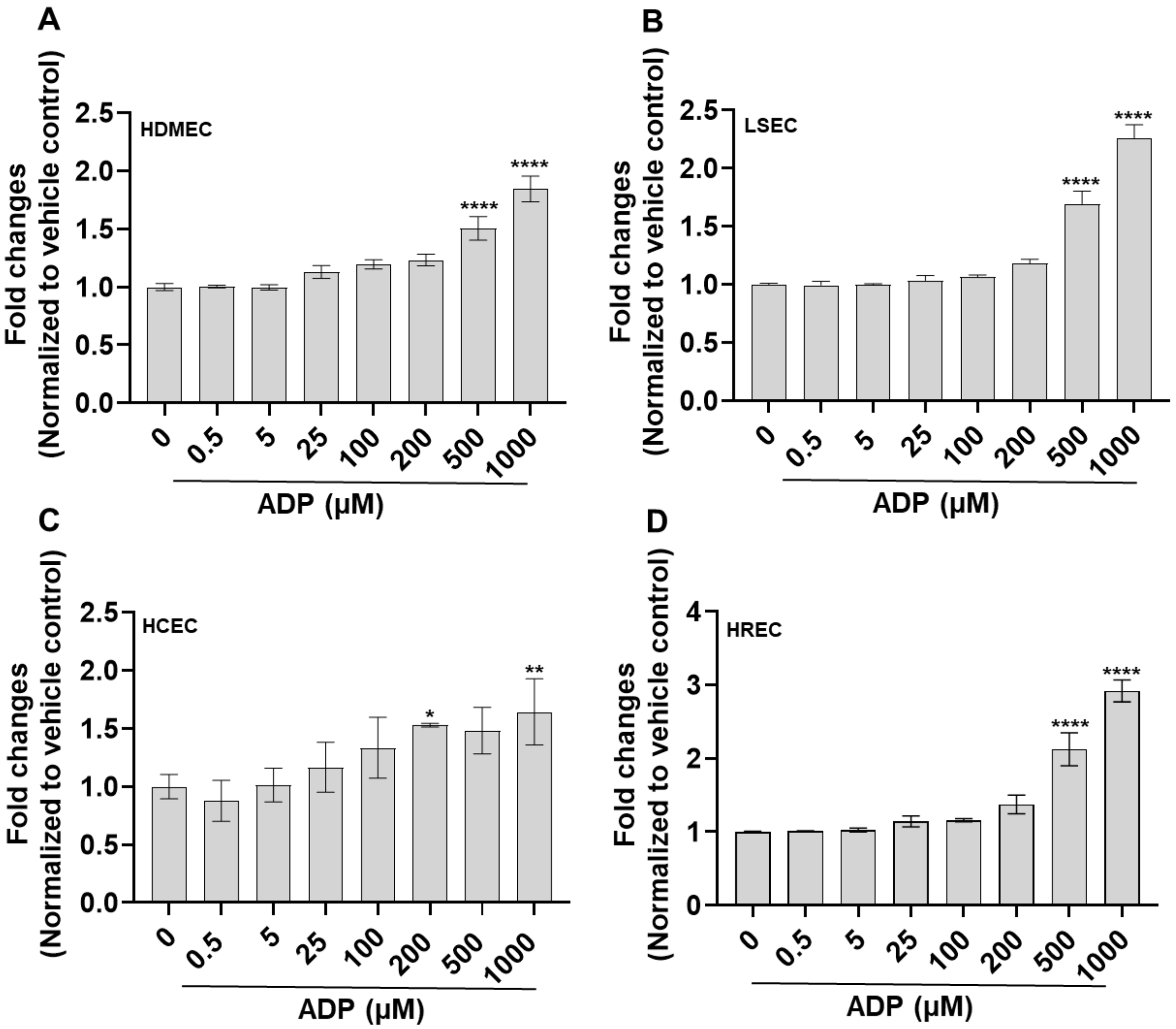

Figure S5. **ADP stimulates proliferation of human EC.** (***A***) HDMEC, (***B***) LSEC, (***C***) HCEC, (***D***) HREC. Cells were incubated with the indicated concentrations of ADP for 3 to 5 days, and cell proliferation was determined as described. The results are expressed as fold increases compared to untreated vehicle control. Growth stimulation was observed in serum-free medium. A representative experiment is shown from three independent studies. The results are presented as the mean ± SD, n = 3. Asterisks indicate significance over untreated vehicle control. One-way ANOVA followed by multiple comparisons with Bonferroni's post- hoc test was used to calculate the statistical significance. *P<0.05, ** P< 0.01, ****P<0.0001.

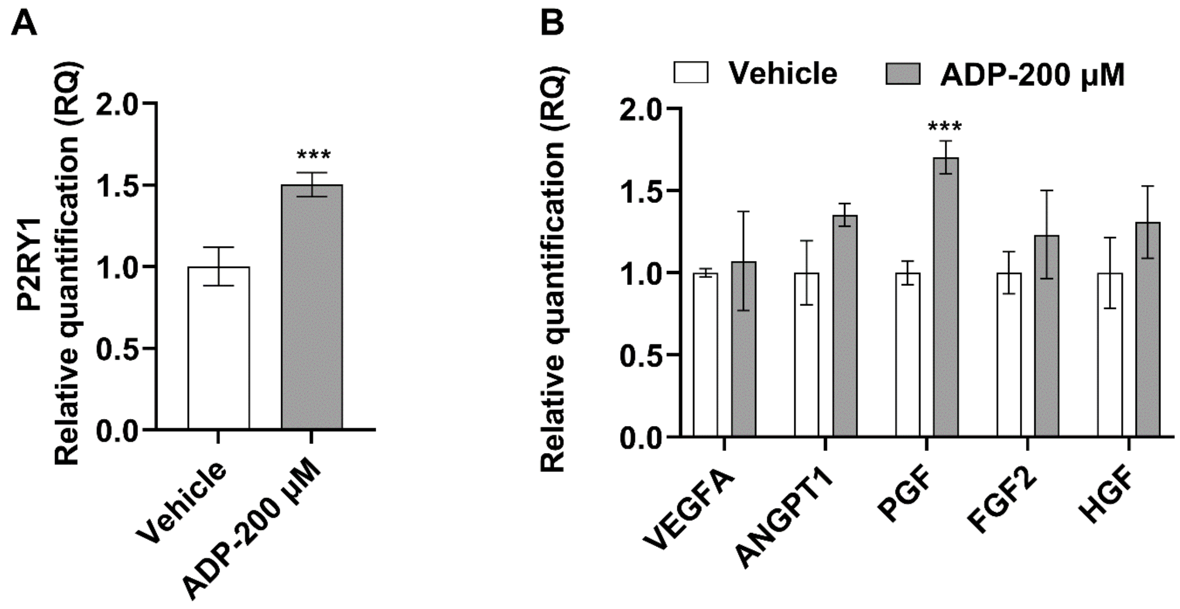

Figure S6. P2Y receptor and growth factors expression in BCEC after ADP treatment. Expression of ADP sensitive receptor (***A***) and growth factors (***B***) was determined by TaqMan qPCR. BCEC were serum-starved, treated with ADP, and harvested 8 hours after ADP addition. ANGPT1, angiopoietin 1; PGF, placenta growth factor; FGF2, fibroblast growth factor 2; HGF, hepatocyte growth factor. A representative experiment is shown from three independent studies. The results are presented as the mean ± SD, $n = 4$. Student *t*-test with Welch's correction and two-way ANOVA followed by multiple comparisons with Bonferroni's correction were used as statistical test. Asterisks indicate significance over untreated vehicle control.

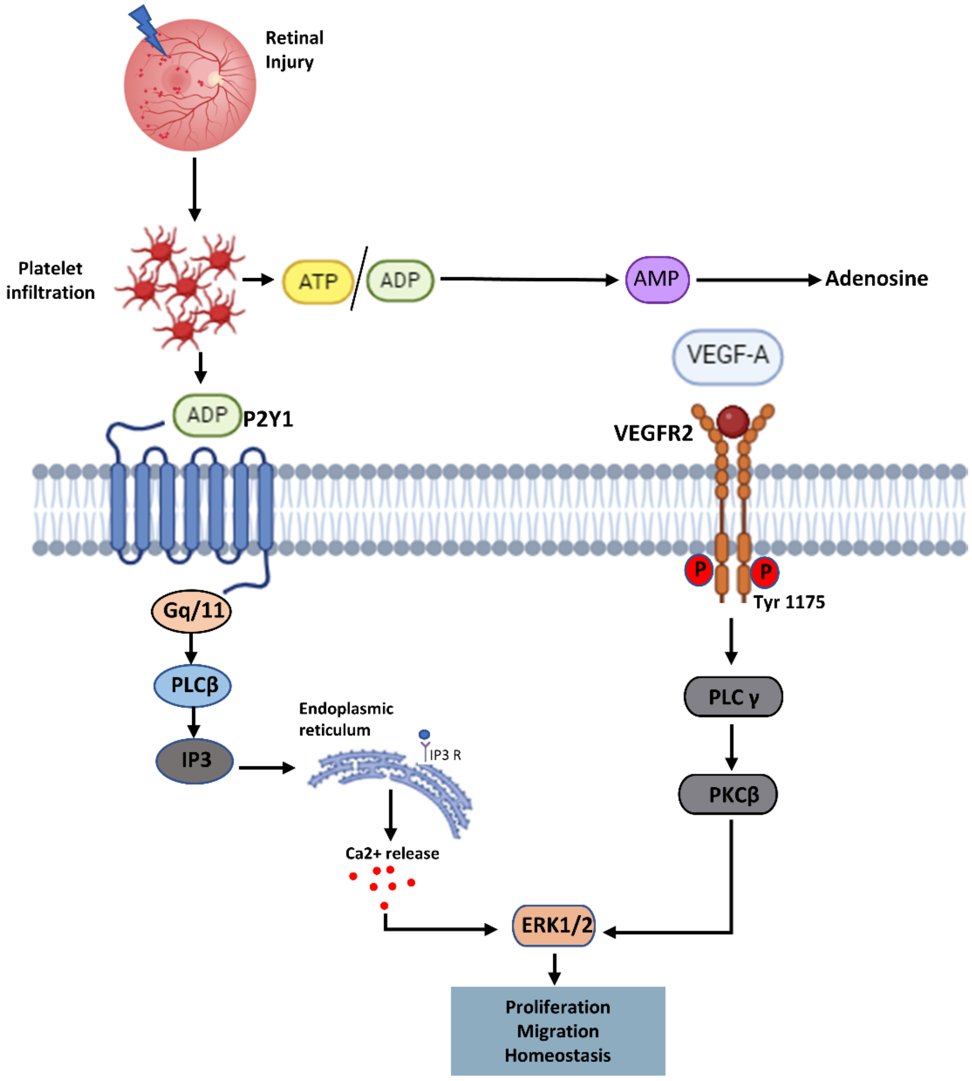

Figure S7. Potential role of ADP and P2Y1 receptor activation in retinal/choroidal angiogenesis. Extracellular ADP, released from damaged tissue or from platelets, binds the G-protein coupled P2Y1 receptor expressed on EC surfaces. Gq/11 -coupled receptor then activates the phospholipase C beta (PLCβ) and inositol triphosphate (IP3) pathway, which lead to the activation of extracellular signal-regulated kinase 1 and 2 (ERK1/2). Ecto-nucleotidases CD39 and CD73, present on the EC surface, can degrade nucleotides and thus act as a negative feedback regulatory mechanism of ADP action on EC. ADP-induced signaling in EC may cooperate with VEGF pro-angiogenic signaling and function in presence of VEGF inhibitors.

SI References

1. H. Xin *et al.*, Heparin-binding VEGFR1 variants as long-acting VEGF inhibitors for treatment of intraocular neovascular disorders. *Proc Natl Acad Sci U S A* **118** (2021).
2. P. Li *et al.*, LIF, a mitogen for choroidal endothelial cells, protects the choriocapillaris: implications for prevention of geographic atrophy. *EMBO Mol Med* **14**, e14511 (2022).
3. C. Zhong *et al.*, Inhibition of protein glycosylation is a novel pro-angiogenic strategy that acts via activation of stress pathways. *Nat Commun* **11**, 6330 (2020).
4. J. C. Garcia-Canaveras, S. Lopez, J. V. Castell, M. T. Donato, A. Lahoz, Extending metabolome coverage for untargeted metabolite profiling of adherent cultured hepatic cells. *Anal Bioanal Chem* **408**, 1217-1230 (2016).
5. M. A. Lorenz, C. F. Burant, R. T. Kennedy, Reducing time and increasing sensitivity in sample preparation for adherent mammalian cell metabolomics. *Anal Chem* **83**, 3406-3414 (2011).
6. T. Bu, S. Kim, Development of metabolome extraction strategy for metabolite profiling of skin tissue. *Metabolomics* **20**, 48 (2024).
7. J. M. Munholland, K. A. Bright, R. N. Nazar, Use of a volatile buffer system in ion-exchange high-performance liquid chromatography of oligonucleotides. *Anal Biochem* **178**, 320-323 (1989).
8. M. A. Strege, S. Stevenson, S. M. Lawrence, Mixed-mode anion-cation exchange/hydrophilic interaction liquid chromatography-electrospray mass spectrometry as an alternative to reversed phase for small molecule drug discovery. *Anal Chem* **72**, 4629-4633 (2000).
9. J. Sofranko, E. Gondas, R. Murin, Application of the Hydrophilic Interaction Liquid Chromatography (HILIC-MS) Novel Protocol to Study the Metabolic Heterogeneity of Glioblastoma Cells. *Metabolites* **14** (2024).
10. C. S. Ho *et al.*, Electrospray ionisation mass spectrometry: principles and clinical applications. *Clin Biochem Rev* **24**, 3-12 (2003).
11. A. P. Voigt *et al.*, Single-cell transcriptomics of the human retinal pigment epithelium and choroid in health and macular degeneration. *Proc Natl Acad Sci U S A* **116**, 24100-24107 (2019).
12. S. Domanskyi *et al.*, Naturally occurring combinations of receptors from single cell transcriptomics in endothelial cells. *Sci Rep* **12**, 5807 (2022).
13. S. Domanskyi *et al.*, Polled Digital Cell Sorter (p-DCS): Automatic identification of hematological cell types from single cell RNA-sequencing clusters. *BMC Bioinformatics* **20**, 369 (2019).
14. V. Lambert *et al.*, Laser-induced choroidal neovascularization model to study age-related macular degeneration in mice. *Nat Protoc* **8**, 2197-2211 (2013).
